# Advocacy in neurology

**DOI:** 10.4103/0972-2327.40232

**Published:** 2008

**Authors:** Apoorva Pauranik

**Affiliations:** Department of Neurology (Medicine), Mahatma Gandhi Memorial Medical College, Indore, India

## Introduction

No man is an island. So are professionals like doctors. Medical persons are primarily expected by themselves and others to be proficient in the theoretical and practical aspects of their subject. However, often it is not realized that they must also be conversant with and be active with regard to many social and political interactions. We live and work in a much larger world. Even though we are proficient in our four primary domains, i.e., clinical, surgical, teaching and research, we encounter situations and moods when we realize that there are many issues that must be changed. At times, there may be the implementation or unwanted changes that we desire to resist. These may be issues related to patient care, our professionalism, clinical practice, competition with other specialties, payments for our performance and protecting the turf or scope of our work.

The importance of advocacy for medical professional is being recognized off late, not the least due to complex social and administrative environment. The latter has a direct effect on the rights and duties of physician, his autonomy and the type of care he is able to provide to his patients. Nothing can be far from the truth that the art and science of medicine is limited to making a diagnosis, writing a prescription or doing a surgery. It is considerably wide and comprehensive. This knowledge is often erroneously assumed to be present *apriori* and no need is felt to define it and describe it in a systemic manner.

### Advocacy regarding what?

Advocacy could be regarding many subjects or issues or situations for which we think that a change or improvement is needed.

The issues could be as broad as the lack of neurologists and neurology residency training programs in country to as narrow and specific as the prevention of peripheral nerve injury by intramuscular injections. The relevance of some issues could be country specific. For example, Medicare and Medicaid payments in USA and tort reforms to cap huge compensation amounts by American courts are not our problems. However, many themes are universal and so are the basic methods of lobbying, pleading, educating, speaking-up and leading [Tables 1 and 2].

**Table 1:** Examples mainly relevant to USA or developed countries

**Issues of concern**	**Actions which may be taken by neurologist**
Better financial reimbursement to neurologists for their cognitive services, which is currently far less in comparison to procedural services	To lobby with insurance firms, third party payers, HMOs about importance of history taking, clinical diagnosis and counseling in comparison to doing to a surgery or angiography or interpreting an MRI.
Escalating insurance premiums for malpractice claims	Public education, Patient safety tips. Tort reforms to prevent frivolous claims and capping the compensation rewards.
Lack of insurance coverage to many patients suffering from neurological diseases	To lobby with insurance company/HMO/ Government.
EMG-NCV being reported by non-neurologists	To lobby with HMOs, insurance company and accreditation councils.

**Table 2:** Examples relevant to India and other developing countries

**Issues of concern**	**Actions which may be taken by neurologist**
Epilepsy was included along with insanity as one of the grounds for annulment of marriage	Senior office bearers of Neurological Society of India lobbied with government, parliament and judiciary to amend that provision
Paucity of neurology and neurosurgery residency training programs and independent upgraded neurology departments in teaching and non-teaching hospitals in public sector all over the country	To increase the awareness on magnitude of disease burden of neurological ailments at community level amongst policy makers, beurocrats, politicians, ministers and media
Poor knowledge of neurological disease amongst general public and patients. Prevalence of myths and stigma and discrimination (e.g., epilepsy)	Public education and patient education initiatives at local individual level as well as collectively at state and national level. This would cover print and electronic media both
Poor up-gradation of basic neurology and neurosurgery knowledge amongst general practitioners and primary health care workers in rural and remote areas	Brief neurology educations programs (CMEs) for such groups at local level in an adhoc manner or at state and national levels in a systematic organized manner
Only a very small fraction of patients with stroke receiving emergency treatment	Public awareness on warning symptoms of brain attack and facilities for stroke units
Poor rehabilitation facilities for neurohandicapped persons	To lobby with state and central governments to establish better departments for physiotherapy, occupation therapy and sheltered workshops at all district hospitals
Lack of patient support groups dedicated to specific neurological diseases	A few organizations have come up in metros and big cities, but more efforts are required

Currently, we have only one neurologist for one million population in India. The number of residency training programs and neurologists being trained each year is highly inadequate. Certain parts of country have lagged behind more than others. A few neurologists have been struggling hard with ministers, secretaries and directors, trying to convince them about the need for the establishment and development of independent neurology and neurosurgery departments in various medical colleges; they require help.

Neurologists are often justly concerned that their professional skills, experience and time spent with patients are not adequately reimbursed. In countries, where payments to physicians are calculated through an extensively predetermined code, sufficient consideration is not given to cognitive services and counseling. Relatively more remuneration is earmarked for procedures and investigations. The associations of neurologists have been constantly working on these matters. The lobbying is likely to be more successful if in addition to office bearers, ordinary members - who have been trained in skills of advocacy and activism - also join in at myriad fronts. In India, the payments are mostly out of packet by patients but things may change in future.

There are other issues pertaining to protecting and expanding the turf of medical practice. For example, should physiotherapists or physiologists be permitted to perform EMG and NCV tests? No, they should not. Should neurologists not get share in reporting on neuroimaging because they provide crucial inputs on clinical condition of patients? Yes, they should. Neurologists need to learn a few lessons from other specialties such as cardiology, radiology and neurosurgery. If cardiologists can do coronary angiography, angioplasty and even carotid artery procedures, why do neurologists fail to claim the terrain, which should naturally belong to them?

We can also act as seed or catalyst or motivators for inception and early groups of patient support growth dedicated to individual neurological diseases in large number of cities and regions. Somehow, we Indians are not good at “joining in” and “being activist,” barring religion or caste based associations. Some neurologists in few metro cities have taken a lead; however, the over all efforts and accomplishment are far too infrequent and too little.

A district collector or divisional commissioner may be approached to provide governmental logistic support for health camps dedicated to one or more neurological diseases. A civil surgeon or district health officer may be requested to propagate health education posters on epilepsy or stoke through the dispensaries in the region.

State and national level quiz competitions with attractive prizes and booklets (which could be carried home) on questions and answers with explanations can increase awareness and popularity of neurology in undergraduate and postgraduate students.

Short-duration certificate courses in neurology for MBBS and MD (medicine) doctors as primary care physicians, family physicians, general practitioners and general duty medical officers may be organized at regional or state levels.

A few years back, a self-proclaimed practitioner of Ayurveda at Rishikesh declared himself as a savior of patients with epilepsy through an expensive advertisement campaign. As a rationale and anticipated act of advocacy and leadership, the neurological community of India should have taken up a united stand and acted against such quacks who make claims at multiple fronts or forums such as media, executive and judiciary.

The use of media in print and electronic format is an essential component of any advocacy effort. A 30 second spot on radio or television that extols the advantages of iodized salt is addressed to general public. It goes a long way in reducing the incidence of a common preventable neurodevelopmental condition with mental retardation. Indian Academy of Neurology should have and could have taken initiative long back to create and launch similar spots for treatability of epilepsy, warning symptoms of brain attack, prevention of head injury by wearing crash helmets, etc.

Public education and patient education overlap but they are distinct activities. Public education is addressed to population at large in a mass manner. It is addressed to every body whether or not affected by that disease. Its core messages are crisps, brief and salient. Rotary District 3040 launched a massive public education campaign on epilepsy in 1990s in 20 districts of western Madhya Pradesh with the help of an educational grant from International League Against Epilepsy. Patient education is more focused and detailed. It can relate to common as well as very rare disorders. It is not distributed randomly in a wide manner but channeled selectively to persons suffering from specific ailments, to patients, their family members and supporting volunteers.

Neuroscientists have great responsibility in constantly being vigilant about the ways and manners in which media portrays myths and negative images about neurological diseases. We must engage them, point out the fallacies and educate them in a decentralized manner by individual neurologists and also in a centralized manner through our state level and national level associations.

More regular, frequent and formal meetings with schoolteachers should be organized in a widespread manner to propagate messages about epilepsy, learning disability (dyslexia), etc.

The code of good practice for employability of persons with epilepsy and other neurological impairments should be popularized amongst employers and human resources managers.

### Advocacy by whom?

From time to time, medical doctors, including neurologists have been playing roles of advocates, leaders or activists, *albeit* mostly in their personal capacity. It has often been considered as a deviation from primary professional duties and rather looked down upon as something nonacademic, inferior or secondary; it need not be the case.

Lobbying, pleading or advocacy can be performed by individuals (neurologists), city-based groups, state level societies, groups formed as per interest (for example, Stroke-subgroups) and national associations. Even international bodies undertake such initiatives. International league against epilepsy launched an ambitious program in 1997 called “out of shadows;” various national societies joined in. This was a classical and excellent example of advocacy. More such examples are required at various levels for stroke, neuromuscular disorder, Parkinsonism, dementia, multiple sclerosis, neurotrauma, brain tumors, developmental disorders, etc. On many occasions, we need and seek and obtain help from nonprofessional organizations. Rotary International has been a major partner in the drive for the eradication of one of the commonest neurological diseases of yesteryears - poliomyelitis.

Neuroscience India Group (Chennai) has been organizing conclaves on to bring out white paper on disability of epilepsy and to change health policy so that patients with refractory epilepsy may get benefits and privileges as disabled persons.

Several nongovernmental organizations can be engaged and co-opted by neurologists in individual capacity at the local level. However, it will help if the regional or national associations evolve a vision, policy, action plan and guidelines.

The American Academy of Neurology and many other national associations have been doing a host of activities that would fall under the umbrella of advocacy - maintaining a website for general public, free distribution of patient education magazines, publication and wide-dissemination of a large variety of brochures, booklets, posters. The Indian Academy of Pediatrics has been organizing quiz for undergraduate medical students at multiple levels with a grand finale during their annual national conference. While waiting for the national neurological bodies to do something similar, Madhya Pradesh Neurological association has taken the lead.

The local branches of Rotary, Lions, Red Cross and other social organizations often pitch in for projects undertaken by neurologists in individual capacities. The Rotary Club of Indore Uptown supported activities on epilepsy, dyslexia, cerebral palsy and stroke.

We need to appreciate that our students and patients can be our best companions in our efforts. It makes greater impact in the minds of people who matter if we substantiate our pleas with real-life stories of patients and secures their vocal support. We shall consider having arrived when we are able to setup a national coalition of a number of patient support groups for as many neurological diseases in as many cities possible. The activism arising from amongst patients shall be the most powerful voice in favor of neurological sciences.

## To Whom? Who are Targets of Advocacy?

Advocacy can be targeted to patients in general when we educate them about safety tips, compliance, follow-up and health-literacy.

Various governments, their departments and institutions are the obvious and common targets of advocacy action at national, state and local levels. Special interest groups approach to even international organizations. In the field of HIV-AIDS, such groups have been highly successful in highlighting issues involved and changing the course of action. On the behest of World Federation of Neurology, International Bureau of Epilepsy (IBE) and International League Against Epilepsy (ILAE), World Health Organization has lately accorded some priority to neurological diseases including stroke and epilepsy.

The American Medical Association and American Academy of Neurology have opposed moves by Federal administration to curb stem cell research on religious grounds.

Judiciary is a very important institution that can play a constructive role if approached in a right, timely and professional manner. The Neurological Society of India lobbied hard in 1980s with the parliamentarians and judiciary for amendment in law pertaining to epilepsy, marriage, divorce and its fallacious equivalence with lunacy or insanity. Public interest litigations are a powerful tool, more so in India where executive arm of government often does not live up to its expectations. Better staffing of independent department of neurology and neurosurgery at medical college Gwalior could be achieved with the help of public interest litigation in Madhya Pradesh High court. It is good that mental health has off late received some due recognition by W.H.O, Government of India and Supreme Court of India. Neurology and Neurosurgery are a part of neurosciences along with psychiatry. We need to do a lot of lobbying at various levels to ensure similar support for our discipline. If our cause is helped by clubbing of neurology with psychiatry or mental health (a sort of piggyback), we need not feel shy or belittled.[1]

Loksabha and Vidhansabhas are empowered to enact laws, many of which have bearing upon neurological practice and patient welfare. Our societies and associations need to oppose or support proposals in legislature depending on situation. Indian Academy of Neurology should attempt to push for tabling and passage of acts mandating minimum supportive treatment for epilepsy at primary care level and basic rehabilitative services for stroke survivors at district level. We have not tapped the avenue of meeting and influencing members of Loksabha, Rajya Sabha and legislative assemblies in states. Imagine the scenario that a large number of our members, having been trained and motivated in advocacy, are constantly meeting representatives, teaching them about various issues that we consider important, reminding them again and again, persuading them to take action by various means such as tabling motions in the house and asking questions to governments. The fact that many elected representatives are not educated or politically mature should not be an excuse.

### Role of formal training

Is it possible to impart formal training to a doctor or for that matter any individual or group so as to make them more efficient advocate of their own and their patients? Will that training result in better orientation and channelization of expertise of doctors with respect to social and political aspects of their work? Will it ultimately benefit the target groups and society as mentioned above? Will it make a difference if professional organization of doctors has a subsection or a cell dealing with issues, which require leadership, lobbying, advocacy or activism? In my opinion, the answer to all the above mentioned questions is an emphatic “yes.” We do need to sensitize our members and officer bearers about various issues that concern and agitate us. Those of us who feel strongly about an issue or other will be able to undertake some action in a better manner if they are helped by a formal training and joining hands with like-minded colleagues.

A policy of lassies-faire is no good. We should not let the things merely drift the way they have been going till now. We should have the confidence of being able to do something positive about our subject, discipline, profession and patients at community level and also at national level. Every neurologist/neurosurgeon has one or more issues that he or she considers important. In an informal postal survey of members of Indian Academy of Neurology and Neurological Society of India, we received around 100 responses. Almost of all agreed about the need for more proactive advocacy by all of us and importance of a formal structured training for the same (Appendix).

Many professional medical organizations have been consistently encouraging, supporting and training their members to become better and more active advocate. Leading in this respect are Accreditation Council for Graduate Medical Education (ACGME) in USA for pediatrics.[2,3] Family practice, orthopedic surgery, ophthalmology and cardiology are other branches, which have developed good programs for organized professional advocacy. Unfortunately such training is still not incorporated at the residency level.

Donald M. Palatucci Forum on advocacy and leadership by American Academy of Neurology is an award-winning program. Many international delegates (including four from India, two from Pakistan) have attended it and vouch for its relevance in our context too.

Palatucci program is an intensive workshop over four days. Thirty trainees are called in after a competitive selection based on narrative answers to questions such as your previous work, your intentions for future, etc. The faculty comprises senior administrative staff from American Academy of Neurology, management gurus, communication experts and leaders from other organizations. Apart from lectures, more emphasis is given to small group discussions, mock rehearsals on making a presentation, meeting a minister, giving an interview, etc. Ten mentors or advisors are called in from amongst previous years graduates who did well. They sit through with new trainees allotted to them and guide them chalking out one action plan each, which will be carried out by that trainee over next one year.

Wasay and Hauth demonstrated the success of the program.[4,5] They analyzed the outcome and impact of the forum through an email questionnaire to 79 odd trainees. The number of hours spent each month on advocacy related activities and accomplishment of action plan goals were the outcomes. For approximately 78% of the advocates, the length of time they spent on advocacy more than doubled after the training. Majority also reported partial or full accomplishment of goals.

Mohammad Wasay (Pakistan) has extensively promoted better neurological healthcare and raised awareness about the burden of neurological conditions in Pakistan. He is leading the government effort to eradicate rabies and tetanus in Karachi and created a stroke support organization and public education campaign. Wasay also developed an advocacy-training program for Pakistani neurologists, that mirror much of what he learned through the Palatucci Advocacy Leadership Forum.

Olga Hardiman (Ireland), launched the Neurological Alliance of Ireland, a nationwide coalition of patient advocacy groups and physicians and authored Standards of Care, the “blueprint” for the development of neurological services in the coming years. Hardiman's effort in unifying neurology professionals to improve patient access to neurological care have included a meeting with the Irish Deputy Prime Minister and Minister for Health.

Wolfgong Grisold (Vienna, Austria) championed a new, annual leadership meeting of the neurological departments of Austria. The first meeting addressed neurology issues in Europe, developments in Austrian Health Policy and neurorehabilitation.

The Indian Academy of Neurology must have an in-house program to train and encourage members interested in advocacy. It will make more than a tangible difference for the better if professional organizations accord due priority to advocacy, leadership and lobbying. A website will be a powerful tool wherein like-minded activists could share views and opinions. An annual training program may be conceived to enhance the skills of interested and motivated members. Trainers can be drawn from fields of business management, media and NGOs. Trainees will be advised to work upon an action plan at a time and chart its progress in a time-bound and measurable manner. Other national associations have been doing good work in India and abroad. There is no need to re-invent the wheel. We may emulate other's plans, with modifications if necessary.

A formal training and interaction with neurologists engaged in some sort of advocacy will boost the morale of members. There are a large number of tips and advices that must be learned and internalized. To be a better advocate one has to shed inhibitions while meeting the officials, politicians, judges, lawyers, police officers, ministers and journalists. It helps if you receive a bit of training for engaging the media, giving interview, submitting memorandum, using advertisement and propaganda, addressing small or large groups.

All of us do act as an advocate of someone or ourselves, sometimes in our life. We need to realize that neurologists can play a very useful role as advocates for the profession of neurology and its beneficiaries, i.e., patients suffering from neurological diseases. If we do not educate the public and governmental officials with regard to who we are and what we do, we risk losing the care that we provide our patients. We have to learn the skills to promote the field in order to improve neurology care that is so badly needed in this country.

## Appendix

**Many issues considered as important by neurologists in a postal survey (100 responders out of 1000 mailings)**

1. National level laws for prevention of head injuries - helmet, seat belt, traffic rules, roadworthiness, licenses, highway patrols, registry, etc. 2. National trauma network. 3. Regional spinal injury centers. 4. “My right to a foot path.” 5. Driving regulations for people with epilepsy. 6. Regional level meetings and programs for advocacy. 7. Accreditation Council for Graduate Medical Education (ACGME) like institution for neurology education. 8. Neurology services at district hospitals. 9. Neurology education in non DM institutions by neurologists. 10. Interventional training for neurologist. 11. Containment of cost of drugs for epilepsy/stroke. 12. More universal medical insurance covering neurological diseases. 13. More subgroups or specialties with in neurology. 14. Neuroepidemiological data collection. 15. Guideline development on neurotuberculosis. 16. Biomedical engineering departments for indigenous designing, development and manufacture of medical, surgical devices. 17. Unethical prescribing practices by doctors - expensive placebo. 18. Increase cooperations amongst local neurologists so as to not to speak ill about colleagues. 19. To discourage off label use of drugs. 20. Brain att ack awareness. 21. Patient support groups. 22. Patient education. 23. Epilepsy education program in schools. 24. To educate public when to go to a neurologist and avoid going wrongly to general practitioners, surgeons, psychiatrist, eye doctors. 25. National epilepsy control program. 26. To influence the media-not to glorify alcohol consumption, smoking, high speed driving. 27. Geriatric neurology care. 28. Guidelines for the management of post head injury neuropsychological complications and assessment of disability for compensation. 29. Definitions, quantitative and qualitative assessment of neurological disability, handicap and impairment. 30. Aphasia assessment and therapy material in Indian languages. 31. Public hygiene - clean India campaign - cysticercosis, Japanese Encephalitis, Neurocritical care society.
